# Glutathione and growth inhibition of Mycobacterium tuberculosis in healthy and HIV infected subjects

**DOI:** 10.1186/1742-6405-3-5

**Published:** 2006-02-20

**Authors:** Vishwanath Venketaraman, Tatanisha Rodgers, Rafael Linares, Nancy Reilly, Shobha Swaminathan, David Hom, Ariel C Millman, Robert Wallis, Nancy D Connell

**Affiliations:** 1 Division of Infectious Diseases, UMDNJ-New Jersey Medical School, Newark, NJ 07103, USA; 2 Center for Emerging and Re-emerging Pathogens, UMDNJ-New Jersey Medical School, Newark, NJ 07103, USA; 3 National Tuberculosis Center, UMDNJ-New Jersey Medical School, Newark, NJ 07103, USA; 4 Department of Medicine, UMDNJ-New Jersey Medical School, Newark, NJ 07103, USA; 5 Department of Microbiology and Molecular Genetics, UMDNJ-New Jersey Medical School, Newark, NJ 07103, USA; 6 New Jersey Medical School, UMDNJ-New Jersey Medical School, Newark, NJ 07103, USA; 7 PPD, 1213 N Street NW, Apt. A, Washington DC 20005, USA

## Abstract

Intracellular levels of glutathione are depleted in patients with acquired immunodeficiency syndrome in whom the risk of tuberculosis, particularly disseminated disease is many times that of healthy individuals. In this study, we examined the role of glutathione in immunity against tuberculosis infection in samples derived from healthy and human immunodeficiency virus infected subjects. Our studies confirm that glutathione levels are reduced in peripheral blood mononuclear cells and in red blood cells isolated from human immunodeficiency virus-infected subjects (CD4>400/cumm). Furthermore, treatment of blood cultures from human immunodeficiency virus infected subjects with N-acetyl cysteine, a glutathione precursor, caused improved control of intracellular *M. tuberculosis *infection. N-acetyl cysteine treatment decreased the levels of IL-1, TNF-α, and IL-6, and increased the levels of IFN-γ in blood cultures derived from human immunodeficiency virus-infected subjects, promoting the host immune responses to contain *M. tuberculosis *infection successfully.

## Introduction

Tuberculosis (TB) is a major global health problem [7]. Approximately one-third of the world's population is latently infected with *Mycobacterium tuberculosis *(LTBI). Individuals with LTBI have a 5–10% lifetime risk of developing active disease [7]. Human immunodeficiency virus (HIV) infected subjects with LTBI are at very high risk of developing active tuberculosis. Development of active TB in HIV patients is due not only to reactivation of latent *M. tuberculosis *infection but also due to increased susceptibility to primary progressive *M. tuberculosis *infection [7].

Innate and adaptive immune responses are required for successful control of *M. tuberculosis *infection. Macrophages provide first line defense against *M. tuberculosis *infection. Murine macrophages can be activated to kill intracellular *M. tuberculosis *by treatment with LPS (a stimulus for TNF-α expression, via triggering of toll-like receptors) and IFN-γ (a product of activated lymphocytes). Nitric oxide (NO) produced by infected macrophages is the main mediator (effector molecule) in this process. Like those of mice, human macrophages also acquire antimycobacterial activity through IFN-dependent interactions with lymphocytes [12]. However, exogenous IFN-γ does not enhance the mycobactericidal activity of isolated human macrophages as it does those of mice. Several studies indicate instead that direct cellular contact is required for the induction of antimycobacterial activity in human macrophages [6,33], and that this activity reflects caspase-mediated induction of apoptosis, triggering of toll-like receptors, the release of antibiotic peptides (e.g., granulysin), or unknown mechanisms [4,36].

Glutathione (GSH) is an antioxidant and plays a vital role in cellular detoxification and enhancement of immune functions [10]. Interestingly, HIV-infected people have subnormal GSH levels in their plasma, lung epithelial lining fluid, peripheral blood mononuclear cells (PBMC), and other blood cells [5,11,14,23]. It has been recently reported that the decreased GSH levels in PBMC of HIV-infected individuals is associated with a poorer prognosis [24]. Immunodeficiency due to HIV-1 represents the greatest recognized threat to successful containment of latent *M. tuberculosis *infection. The aim of this study was to examine the role of GSH in immunity against TB in samples derived from healthy and HIV infected subjects.

In our previous studies using macrophages from different sources, we have demonstrated that GSH plays a vital role in innate immunity against TB infection [40,41]. In our recent studies we have shown that GSH has static effect on H37Rv growth *in vitro *[41]. The mechanism of toxicity of GSH to mycobacteria is not yet known. One possibility is that the presence of high concentrations of GSH may result in an imbalance in a bacterial cell already containing an alternative thiol for regulating reduction/oxidation activity (e.g., mycothiol).

In the present study, we reexamined the extent to which GSH levels are decreased in HIV positive subjects. We also examined the relationship between GSH levels and the ability to kill intracellular *M. tuberculosis*, in association with other immune functions, such as cytokine production. GSH levels were modulated by treating blood samples with N-acetyl cysteine (NAC) to increase or buthionine sulphoximine (BSO) to decrease intracellular GSH pools. Our results suggest that the inability of immune cells from healthy and HIV subjects to contain TB growth may be a consequence of the inability of their macrophages to maintain adequate GSH levels during *in vitro *infection.

## Experimental methods

### Subjects

A total of 20 subjects (10 healthy volunteer controls and 10 patients with HIV infection) were enrolled at UMDNJ-University Hospital of Newark and the NJ Medical School, in Newark, NJ. Subjects with HIV infection without tuberculosis (n = 10) were recruited at the Infectious Disease Clinic of UMDNJ-University Hospital. The Clinic is the site of several ongoing studies of HIV treatment; these studies provide anti-retroviral treatment (ART) to enrolled subjects without charge. Patient care was not altered by participation in this study. Patients were defined as being HIV-positive on the basis of a positive ELISA with a confirmatory Western Blot performed as part of their routine care in the clinic. The average CD4 numbers for HIV patients in this study was 423 ± 83/cumm. Only one patient had CD4 counts below 200/cumm. Seven patients were on ART and three patients were not on any treatment at the time of blood draw. Healthy subjects without HIV infection or a history of TB were recruited from the hospital and the university faculty and staff (n = 10). Healthy and HIV-positive subjects with a history of a positive tuberculin test (TST) were excluded from the study so as to maintain strict study definitions. This is according to the CDC recommendation that recognizes that a positive TST reflects latent TB infection.

### Safety precautions for handling *M. tuberculosis*

All experiments with *M. tuberculosis *H37Rv were performed inside the bio safety level 3 (BSL-3) facility. The protocols for all experiments were approved by the UMDNJ Institutional Review Board, and the New Jersey Medical School Institutional Biosafety Committee. All experimental procedures were performed inside the biosafety cabinets in the BSL-3. All liquid and solid wastes from the experiments were treated with a disinfectant solution and then autoclaved.

### Processing of H37Rv for infection

*M. tuberculosis *H37Rv was grown in 7H9 with albumin-dextrose complex (ADC). Static cultures of mycobacteria at peak logarithmic phase of growth (between 0.5 and 0.8 at *A*600) were used for infection. The bacterial suspension was washed and resuspended in RPMI containing AB serum. Bacterial clumps were disaggregated by vortexing five times with 3-mm sterile glass beads. The bacterial suspension was passed through a 5 μm filter to remove any further clumps. The total number of organisms in the suspension was determined by plating. Processed mycobacteria were frozen as stocks at -80°C. At the time of infection, frozen stocks of processed mycobacteria were thawed and used for macrophage infection.

### Separation of monocytes from human blood

Human monocyte-derived macrophages (HMDM) were used to study the effects of IFN-γ and GSH in inducing intracellular killing of H37Rv. These experiments were performed only in blood samples from healthy subjects due to the non-availability of sufficient blood volume from HIV patients. Forty ml of blood from healthy subjects were used for monocyte isolation. PBMC were isolated by ficoll hypaque density centrifugation. PBMC were washed with PBS and resuspended in RPMI containing 5% AB serum. PBMC (10 × 10^6^/ well) were distributed into Poly-DL-lysine coated 12 well plates and incubated overnight at 37°C, 5% CO_2 _in a humidified atmosphere, to allow monocytes to adhere to the plate. Non-adherent cells were removed by gentle washing and the adherent monocytes were cultured in RPMI containing 5% AB serum for 7 days before being used for infection experiments to allow differentiation to macrophages. The total number of macrophages per well (on day seven) was quantitated by detaching the macrophages from a single well by the addition of ice-cold accutase (Sigma). Viable detached macrophages were counted in a Neubauer counting chamber by trypan blue dye exclusion. The average number of macrophages per well on day 7 is approximately 5 × 10^5^.

### Macrophage infection

HMDM from healthy subjects were maintained *in vitro *as described above. Macrophages were infected with processed H37Rv at moi of 10:1. Macrophages were incubated with H37Rv for 2 h (for phagocytosis), after which extracellular organisms were removed by washing with PBS. Infected macrophages were maintained in RPMI containing 5% AB serum. Infected macrophage cultures were terminated at 4 h and 7 days after infection and treatment, to measure the intracellular viability of H37Rv. Cell free supernatants from infected macrophage cultures were diluted and plated for extracellular bacterial growth. Intracellular viability of H37Rv was determined by lysing the infected macrophages with sterile distilled water and plating the lysate on 7H11 enriched with ADC, to enumerate mycobacterial colonies.

### Survival of H37Rv in IFN-γ, LPS treated HMDM

IFN-γ is considered a predominant activator of microbicidal functions in macrophages and is essential for prevention of uncontrolled progression of *M. tuberculosis *infection [2,18,27]. We therefore studied the survival of H37Rv in IFN-γ, LPS treated HMDM. HMDM were maintained *in vitro *and infected with H37Rv, as described previously. H37Rv-infected HMDM were treated with IFN-γ (100 U/ml) and LPS (1 μg/ml), the cultures were terminated at 4 h and 7 days after infection and treatment, to determine the intracellular viability of H37Rv inside unstimulated and IFN-γ, LPS-stimulated macrophages.

### Survival of H37Rv inside NAC treated HMDM

We determined the effects of GSH in human macrophage mediated growth inhibition of intracellular H37Rv. HMDM were treated with different concentrations of NAC. Cysteine uptake is considered as rate-limiting step for synthesis of GSH. The most efficient way to increase the levels of cysteine in cells grown *in vitro *is to supply the culture medium with NAC. NAC is easily taken up by the cells and is non-toxic. Intracellularly, NAC is de-acetylated and cysteine is utilized for GSH synthesis. H37Rv infected HMDM were treated with 5, 10, 15, and 20 mM NAC, and intracellular growth of H37Rv was studied. Infected macrophage cultures were terminated at 4 h and 7 days, after infection and treatment. Infected macrophages were lysed and plated for mycobacterial colonies.

### Whole blood mycobactericidal assay

Mycobacteria added to heparinized blood (after dilution with tissue culture medium), are rapidly ingested by monocytes and other phagocytic cells such as neutrophils. This model differs from other intracellular infection models in that all blood elements are represented. Interactions of infected monocytes with natural killer cells and antigen-specific T cells result in control of intracellular growth. In contrast to the studies with isolated macrophages, the whole blood assay requires a low volume of blood. Blood was diluted at the following proportion: 300 μl of blood from healthy subjects and patients were diluted to 1 ml with RPMI. Blood cultures were infected with 10^5 ^CFU of H37Rv. GSH levels in blood cultures were altered using agents such as NAC (10 mM) and BSO (500 μM) that specifically increase and decrease intracellular GSH. The effect of altered GSH levels on *M. tuberculosis *survival was studied. Treatment of cells with BSO causes inhibition of GSH synthesis. BSO specifically inhibits the activity of γ-glutamyl-cysteinyl synthetase enzyme, that catalyses the first step reaction in the synthesis of GSH. Blood cultures were treated with either NAC or combination of NAC and BSO for 24 h prior to infection. H37Rv infected whole blood cultures were incubated at 37°C and harvested at selected intervals (2 h and, 48 h) by sedimentation at 2000 rpm for 10 min. Supernatants were used to determine cytokine levels and extracellular mycobacterial growth. Host cells were disrupted by addition of sterile water. The lysates were plated on 7H11 medium enriched with ADC for mycobacterial colonies.

### Assay of GSH

Intracellular GSH levels in PBMC, red blood cells (RBC), and plasma, from healthy individuals and HIV positive subjects were assayed by spectrophotometry, using a GSH assay kit procured from Calbiochem. This approach is used to determine whether GSH levels are decreased in all blood components or just in some specific components. Plasma and cell lysates of RBC and PBMC, derived from healthy and HIV positive subjects, were mixed with equal volume of ice cold 5% metaphosphoric acid (MPA) and centrifuged at 3000 rpm for 15 minutes. Supernatants were used for GSH assay, as per the manufacturer's instruction. Plasma, RBC, and PBMC were separated from whole blood by density gradient centrifugation using ficoll hypaque. Samples were also used for protein assay by Bradford's method using Bio Rad reagent.

### Cytokine assay

Blood cultures were prepared by afore mentioned methods. Blood cultures from healthy subjects and HIV patients were treated as follows: no treatment, infection with H37Rv, and infection with H37Rv and treatment with NAC. Cultures were terminated at 2 h and 48 h, after infection. Uninfected cultures were terminated at the same time points. Cultures were centrifuged at 2000 rpm for 10 min. Cell free supernatants from healthy and HIV patients were used for the cytokine assay, which was performed using a Beadlyte kit procured from Upstate. This is a highly sensitive kit that can be used to detect multiple cytokines in tissue culture samples. A monoclonal antibody specific for a cytokine is covalently linked to a fluorescent bead set, which captures the cytokine. A complementary biotinylated monoclonal cytokine antibody then completes the immunological sandwich and the reaction is detected with streptavidin-phycoerythrin using a Luminex. The assay was performed as per the manufacturer's protocol.

### Statistical analysis

Statistical analysis of the data was carried out using Statview program and the statistical significance was determined using unpaired t test. Data from cytokine assays was analyzed by non-parametric test (Kruskal-wallis). Differences were considered significant at a level of p < 0.05.

## Results

### Survival of H37Rv in HMDM

**Figure 1:**
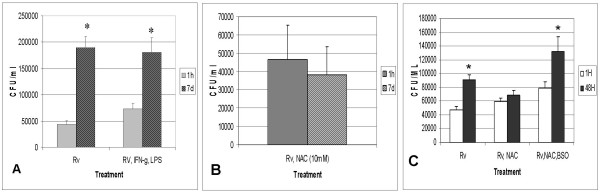
**Growth of H37Rv in unstimulated (Fig 1a), IFN-γ, LPS (Fig 1a), and NAC treated HMDM (Fig 1b)**. Human monocytes from peripheral blood were maintained *in vitro *in RPMI containing 5% AB serum, for 7 days for differentiation to macrophages. HMDM were infected with H37Rv at moi of 10:1 and maintained in media alone (Fig 1a) or in media containing IFN-γ, LPS (100 U/ml and 1 μg/ml), respectively, (Fig 1a) or media containing, NAC 10 mM (Fig 1b). Infected macrophages were terminated at 4 h & 7 d after infection to determine the intracellular growth of H37Rv. Intracellular colony counts of H37Rv were determined by plating lysed cultures on Middlebrook 7H11. Fig 1a, are means from six different experiments performed in triplicate. Fig 1b, are means from three different subjects performed in triplicate. (Fig 1c) **Whole blood Infection of H37Rv**. Blood from healthy individuals was diluted at the following proportion, 300 μl of blood was diluted to 1 ml with RPMI. One milliliter of diluted blood was added to each well of 12 well tissue culture plates. Blood cultures were treated with none or NAC (10 mM) or NAC, BSO (500 μM) for 24 h. Blood cultures were infected with processed H37Rv. Infected blood cultures were terminated at 2 h & 48 h after infection, to determine the intracellular viability of H37Rv. Intracellular viability of H37Rv was determined by plating the diluted blood cell lysates on 7H11. Data in Figure 1c are averages from seven subjects performed in triplicate.

We studied the survival of H37Rv in HMDM from healthy subjects. H37Rv-infected HMDM were treated with IFN-γ (100 U/ml) and LPS (1 μg/ml), and the intracellular viability of H37Rv inside unstimulated and IFN-γ, LPS-stimulated macrophages was compared. Figure 1a shows results from six different subjects performed in triplicate. We observed significant growth of H37Rv inside unstimulated HMDM between 1 h and 7 days (Fig 1a). The increase was almost four fold. Stimulation of HMDM cells with IFN-γ, LPS also resulted in significant growth of intracellular H37Rv (Fig 1a). However, the increase in H37Rv growth was less than three-fold (Fig 1a). To examine whether GSH plays a major role in human macrophage killing of H37Rv, HMDM from healthy volunteers were treated with 5, 10, 15, and 20 mM NAC, and intracellular growth of H37Rv was measured. Experiments performed in six different subjects show that treatment of HMDM with 10 mM NAC resulted in stasis in H37Rv growth in three out of six subjects (Fig 1b). Treatment of HMDM with NAC at 5 mM and 15 mM induced growth inhibition of H37Rv, in one out of six, and two out of six subjects, respectively (data not shown). Treatment with 20 mM NAC had no effect on growth inhibition of H37Rv (data not shown). Therefore, NAC at 10 mM is more effective in inducing growth control of *M. tuberculosis *as compared to IFN-γ, LPS, or other concentrations of NAC (Fig 1b) in isolated HMDM.

### Whole blood model

Several studies indicate that direct cell contact is required for induction of antimycobacterial activity in human macrophages [6,33], and that this activity reflects caspase-mediated induction of apoptosis, triggering of toll-like receptors, the release of antibiotic peptides (e.g., granulysin), or unknown mechanisms [4,36]. Mycobacteria are rapidly ingested by phagocytic cells when added to heparinized blood (after dilution with tissue culture medium). This model differs from other intracellular infection models in that all blood elements are represented.

We therefore tested whether interaction of monocytes with other immune cells will lead to growth inhibition of intracellular H37Rv using whole blood cultures, which provides a micro-environment that is conducive for cellular interactions.

### Whole blood mycobactericidal assay in healthy subjects

Blood from healthy volunteers was diluted as described and treated with none or 10 mM NAC. The blood cultures were then infected with 5 × 10^5 ^CFU of processed H37Rv. Infected blood cultures were terminated at 2 h and 48 h after infection to determine the intracellular viability of H37Rv. Cell suspensions were centrifuged to separate the cell free supernatants and pellets. Supernatants were diluted and plated for extracellular bacterial growth. Intracellular viability of H37Rv was determined by plating the diluted blood cell lysates on 7H11. Infection of blood cultures with H37Rv resulted in almost two-fold increases in the intracellular growth of H37Rv (Fig 1c). The increase in H37Rv growth was statistically significant. Treatment of blood cultures with NAC (10 mM), caused growth inhibition of H37Rv in all seven individuals tested (Fig 1c). The data in Fig 1c are averages from seven healthy subjects. Treatment of cultures with BSO abrogated the growth inhibition effect of NAC (Fig 1c). These results indicate that growth inhibition of H37Rv in NAC treated blood cultures is due to combination of direct antimycobacterial effects of GSH and activation of immune cells induced by GSH.

**Figure 2:**
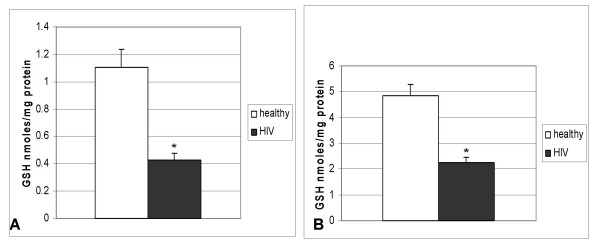
**Spectrophotometric assay of GSH in PBMC (Fig 2a) and RBC (Fig 2b), derived from healthy and HIV positive subjects**: GSH assay kit was procured from Calbiochem. PBMC and RBC lysates (from HIV-infected and healthy subjects), were mixed with equal volume of ice cold 5% metaphosphoric acid (MPA) and centrifuged at 3000 rpm for 15 minutes. Supernatants were used for GSH assay, as per manufacturer's instruction. Samples were also used for protein assay by Bradford's method using Bio Rad reagent.

### Levels of GSH in blood samples from healthy and HIV-positive subjects

Intracellular GSH levels in PBMC and RBC were assayed by spectrophotometry as described. We observed a significant and more than 50% decrease in intracellular GSH levels in PBMC (Fig 2a) and RBC (Fig 2b) from HIV patients compared to healthy subjects. Data shown in Fig 2 are averages from six healthy and six HIV-infected subjects. We observed no difference in the plasma GSH levels between healthy and HIV patients.

**Figure 3:**
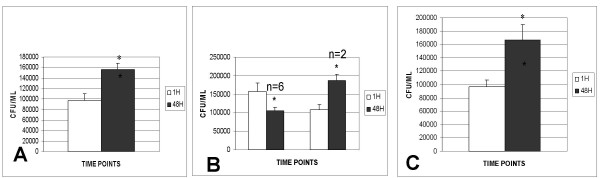
**Growth of H37Rv in whole blood cultures of HIV patients**. Blood from HIV positive subjects was diluted at the following proportion, 300 μl of blood was diluted to 1 ml with RPMI. One milliliter of diluted blood was added to each well of 12 well tissue culture plates. Blood cultures were treated with none (Fig 3a), or 10 mM NAC (Fig 3b) or NAC, 500 μM BSO, (Fig 3c) for 24 h. Blood cultures were infected with processed H37Rv. Infected blood cultures were terminated at 2 h & 48 h after infection, to determine the intracellular viability of H37Rv. Intracellular viability of H37Rv was determined by plating the diluted blood cell lysate on 7H11. Data in Figure 3a, b, and c, are averages from four, eight and three subjects, respectively.

### Growth control of H37Rv by NAC-treated blood cultures from HIV patients

Intracellular growth of H37Rv was monitored in blood cultures of HIV-positive subjects. We observed a significant growth of H37Rv in unstimulated blood cultures (Fig 3a). NAC treatment induced growth inhibition of intracellular of H37Rv. Data in Fig 3b are averages from data obtained from eight different HIV-positive subjects. BSO treatment abrogated the inhibitory effect brought about by NAC treatment (Fig 3c).

**Figure 4:**
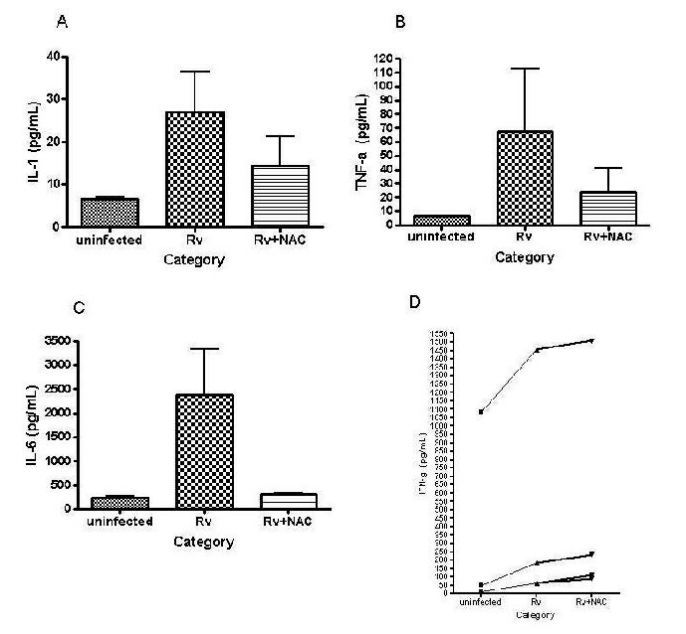
**IL-1, TNF-α, IL-6 and IFN-γ assays in blood culture supernatants**: Blood cultures from HIV patients were treated as follows, no treatment, infection with H37Rv, and infection with H37Rv and treatment with NAC. Cultures were terminated at 48 h, after infection. Uninfected cultures were terminated at the same time points. Cultures were centrifuged at 2000 rpm for 10 min. Cell free supernatants were used for assay of IL-1 (Fig 4a), TNF-α (Fig 4b), IL- 6 (Fig 4c) and IFN-γ (Fig 4d). Cytokine assay was performed using a Beadlyte kit procured from Upstate. The assay was performed as per manufacturer's protocol.

### Assay of cytokines in blood culture supernatants from healthy and HIV-positive subjects

Cytokines were measured in blood culture supernatants from healthy and HIV-infected subjects. Interestingly, in HIV subjects, H37Rv infection induced the blood cultures to produce increased levels of pro-inflammatory cytokines such as IL-1, TNF-α, and IL-6 (Fig 4a, 4b, 4c). H37Rv infection induced almost three fold increases in IL-1 production, compared to uninfected controls (Fig 4a). NAC treatment of H37Rv infected cultures down-regulated IL-1 levels (Fig 4a). Compared to uninfected controls, H37Rv infection induced seven fold increases in TNF-α levels in two patients tested (Fig 4b). NAC treatment of H37Rv infected cultures caused reduction in TNF-α levels (Fig 4b). H37Rv infection induced almost ten fold increases in IL-6 production, in three patients tested (Fig 4c). NAC treatment reduced the levels of IL-6 to those found in the uninfected control. We also observed that infection of HIV blood cultures with H37Rv caused six fold increases in IFN-γ production in two patients and three fold increases in one patient (Fig 4d). In comparison to untreated controls, NAC treatment of H37Rv infected cultures induced ten fold increases in IFN-γ production in two patients and almost four fold increases in one patient (Fig 4d). In summary, our studies show that NAC treatment down-regulated the synthesis of IL-1, IL-6, and TNF-α and increased the levels of IFN-γ (Fig 4a, 4b, 4c, 4d).

**Figure 5:**
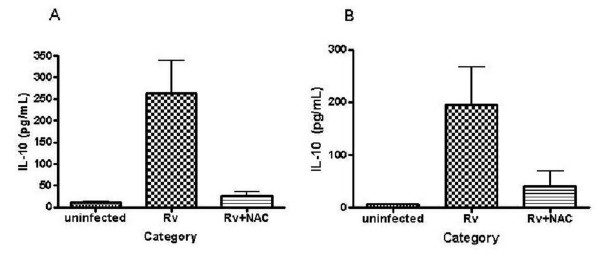
**IL-10 assay in blood culture supernatants**: Blood cultures from healthy subjects and HIV patients were treated as described previously. Cultures were terminated at 48 h, after infection. Cell free supernatants, from healthy (Fig 5a), and HIV patients (Fig 5b), were used for assay of IL-10. Cytokine assay was performed using a Beadlyte kit procured from Upstate. Data in Figure 5a and b are averages from four healthy and five HIV-infected subjects, respectively.

With the exception of IL-10, all other cytokines produced by healthy subjects showed no clear trend. The regulation of IL-10 synthesis in response to H37Rv infection and NAC treatment was similar in healthy subjects and HIV patients. H37Rv induced almost ten-fold increases in IL-10 levels in both healthy and HIV-infected subjects (Fig 5a, 5b). Furthermore, NAC treatment reduced the levels of IL-10 to those found in uninfected controls, in both healthy subjects and HIV patients (Fig 5a, 5b).

**Figure 6:**
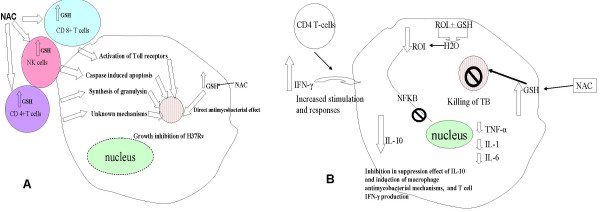
**Model describing direct and indirect effects of GSH in growth control of H37Rv in blood cultures derived from healthy and HIV-infected subjects.** (Fig B) Model describing the effect GSH in modulating cytokine synthesis in whole blood cultures derived from HIV positive subjects.

## Discussion

Development of TB in HIV infected patients is based on a predisposition to reactivation of latent *M. tuberculosis *infection and to susceptibility to primary progressive *M. tuberculosis *infection [9]. However, the relationship of host immune responses to the development of TB during different stages of HIV disease is not clear. The opportunistic behavior of *M. tuberculosis *during human HIV infection can be explained by suppression of type-1 responses at the level of antigen-presenting cells, CD4 T cells and effector macrophages.

*In vitro *studies have shown that lowering of intracellular GSH levels decreases cell survival, alters T cell functions and increases HIV replication, NF-kB activation, and sensitivity to TNF-α induced cell death [10,11,19]. A role has also been proposed for GSH as a carrier molecule for NO. Nitric oxide also reacts with GSH to form GSNO, an NO donor with greater stability [34,35].

We first reported that GSH facilitates the control of intracellular *M. bovis *BCG in NO-deficient macrophages derived from iNOS knock out mice, and in HMDM [40]. These studies indicated that GSH has direct antimycobacterial activity distinct from its role as an NO carrier. Furthermore, in our recent studies we demonstrated that GSH is vital for growth control of intracellular H37Rv in J744.1 macrophages [41].

It has been reported that production of IFN-γ is crucial to the control of *M. tuberculosis *infection [18]. Impaired production of IFN-γ correlates with progression of immunodeficiency and is likely related to abnormalities in the IL-12-IFN-γ axis [8,31]. We therefore tested the growth of H37Rv in HMDM from healthy subjects that are unstimulated or stimulated *in vitro *with IFN-γ, LPS. We observed a significant, four-fold increase in growth of H37Rv inside unstimulated HMDM, between 1 h and 7 days (Fig 1a). Stimulation of H37Rv-infected HMDM cells with IFN-γ, LPS also resulted in a three-fold increase in growth of intracellular H37Rv (Fig 1a). Since our earlier studies suggested a role for GSH in innate immunity against *M. tuberculosis*, we tested whether NAC treatment would induce HMDM to inhibit the growth of H37Rv. We observed that NAC at 10 mM concentration induced growth inhibition of H37Rv in three out of six healthy individuals tested (Fig 1b). Although normal levels of GSH are present in cells derived from healthy subjects, those levels might decrease during oxidative and nitrosative stress generated during TB infection. Therefore, addition of NAC to HMDM caused growth inhibition of *M. tuberculosis *by augmenting intracellular GSH levels. These results suggest that growth inhibition of H37Rv in NAC treated HMDM is due to the direct antimycobacterial effects of GSH. Furthermore, the inability of HMDM from some healthy individuals to inhibit *M. tuberculosis *growth is probably due to the inability of macrophages to maintain adequate GSH levels, despite NAC treatment.

As described before, innate and adaptive immunity are essential for successful elimination of *M. tuberculosis*. Macrophages interact with other immune cells *in vivo*, for successful growth retardation of *M. tuberculosis*. The whole blood model of infection resembles an *in vivo *system in promoting cellular interactions. This model differs from other intracellular infection models in that all blood elements are represented. Infection of blood cultures from healthy volunteers with H37Rv resulted in an almost two-fold increase in H37Rv growth (Fig 1c). The increase in H37Rv growth was statistically significant. In contrast to HMDM, treatment of blood cultures with NAC (10 mM) caused growth inhibition of H37Rv, in all seven individuals tested (Fig 1c). Our results suggest that growth inhibition of H37Rv in NAC treated blood cultures is due to direct antimycobacterial effects of GSH and due to activation of blood cells induced by GSH.

We have confirmed the work of others that GSH levels are decreased in patients with HIV-1 infection [5,11,14,23], and then hypothesized that this decrease would be associated with reduced capacity of monocytes to kill intracellular *M. tuberculosis*. We further proposed that NAC treatment would improve the killing of *M. tuberculosis*. We tested our hypothesis by determining GSH levels in healthy and HIV positive subjects. We observed a significant and more than 50% decrease in GSH levels in PBMC and RBC from HIV patients compared to healthy subjects (Fig 2a, 2b). Since GSH enhances innate and adaptive immune functions, GSH deficiency in PBMC may contribute to the progressive immune dysfunction of HIV infection. Macrophages play a central role in HIV and TB infection because they are among the first cells to be infected [19]. Moreover, macrophages serve as an important reservoir for both HIV and *M. tuberculosis*. The major obstacle to eradication of HIV is latent virus in these reservoirs which has prompted the search for new drugs and strategies to protect this cell compartment. Erythrocytes have been used as a carrier system to deliver antiretroviral molecules to macrophages selectively. Fraternale *et al *[19] have reported that treatment of mice with AZT+DD1+GSH-loaded RBC significantly reduces the proviral DNA content, compared to mice treated with AZT+DD1. This result is consistent with our hypothesis and suggests that low levels of GSH in RBC, as observed in this and other studies, will affect the GSH carrier functions of RBC, compromising GSH delivery to macrophages.

In order to determine the effects of NAC treatment on PBMC and RBC in reducing the growth of intracellular H37Rv, whole blood cultures from HIV patients were treated *in vitro *with NAC and infected with H37Rv. We observed significant growth of H37Rv in unstimulated blood cultures from HIV patients (Fig 3a). *In vitro *NAC treatment to blood cultures derived from HIV subjects caused inhibition in growth of intracellular H37Rv (Fig 3b). Furthermore, BSO treatment abrogated the inhibitory effect brought about by NAC treatment (Fig 3c). This suggests that restoration of GSH levels in HIV subjects caused enhancement in immune cell functions to contain *M. tuberculosis *growth.

The decreased GSH content in immune cells of HIV-positive individuals was atleast in part attributed to the decreased in plasma cysteine and increased plasma glutamate (an inhibitor of cysteine permeation via the Xc- transport system), as observed during early infection. The decreased intracellular GSH and plasma cysteine observed in HIV patients is due to chronic oxidative stress, which may lead to the progression of the disease. The decreased availability of cysteine can be overcome to some extent by the cysteine precursor NAC [13]. A recent report of a carefully conducted clinical trial indicates that NAC treatment improves the clinical situation and delays the HIV disease progression [24]. This study showed that long-term administration of NAC to AIDS patients improves their hematological profile, GSH content and life expectancy [24].

We measured cytokine levels in whole blood culture supernatants from healthy and HIV infected subjects. No clear trend in cytokine profile was observed in healthy subjects. Interestingly, we observed that *in vitro *infection with H37Rv induced the whole blood cultures from HIV patients to synthesize increased levels of cytokines such as IL-1, TNF-α, IL-6 and IL-10 (Fig 4, 5). IL-1, TNF-α, IL-6 are the early pro-inflammatory cytokines produced by monocytes after various bacterial infections and share a wide array of biological activities [4,5]. *In vitro *studies have shown that mycobacterial preparations, including lipoarabinomannan, can cause the release of TNF-α and IL-1 from human PBMC [25,42,44].

The release of pro-inflammatory cytokines after mycobacterial infection is a host immune response that may be propitious or deleterious to the host. Newman *et al*. reported that increased survival of *M. avium intracellulare *(MAI) in isolated macrophages is correlated with the efficiency with which TNF-α and IL-6 are produced in response to MAI infection [28]. Nevertheless, increased levels of these pro-inflammatory cytokines may be disadvantageous to the host because they not only cause acute-phase events, such as fever, but also mediate cachexia, hemorrhagic necrosis and lethal shock [29,30,37]. TNF-α by classical cascade is known to up-regulate the levels of IL-1 and IL-6.

Elevated levels of IL-6 are present in plasma of patients with TB [15]. Studies by Van Heyningen *et al *[39] indicate that macrophages infected with *M. bovis *BCG released copious amounts of IL-6 which in turn inhibited the macrophage capacity to induce proliferation of CD4 T cell hybridoma. Nagabhushanam *et al*. [26] reported a novel function of IL-6 in inhibiting cellular immune response to eradicate *M. tuberculosis *infection. Their studies show that IL-6 produced by *M. tuberculosis*-infected macrophages selectively inhibited macrophage responses to IFN-γ. In other words, secretion of IL-6 by *M. tuberculosis*-infected macrophages may contribute to the inability of IFN-γ to eradicate *M. tuberculosis *infection [26].

The high levels of IL-6 released by infected macrophages have implications for co-infection with HIV [32]. Mycobacterial infections are one of the most common AIDS-defining illnesses and may even accelerate progression to AIDS [17]. The two infections seem to synergize, causing a shift of the host-pathogen balance in favor of the pathogen, which cannot be reversed by treatment with antimycobacterial agents [43].

TNF-α and IL-6, as well as IL-1, can increase HIV replication [3,21]. Thus, decreasing the pro-inflammatory cytokine production *in vivo *may enhance the control of viral replication. Elevated levels of IL-6, TNF-α and IL-10 have been described previously in cases of advanced HIV disease [1,20,22]. Therefore, increases in the levels of pro-inflammatory cytokines will cause a positive feedback loop in which the two infections complement one another, leading to accelerated progression of both diseases.

In our studies, we observed that NAC treatment caused down-regulation of the synthesis of IL-1, IL-6, and TNF-α (Fig 4a, 4b, 4c), and up-regulation of the synthesis of IFN-γ (Fig 4d). These results suggest that GSH might have a crucial role *in vivo *in reducing the levels of pro-inflammatory cytokines thereby protecting the host against disease progression.

Active TB is associated with suppression of T cell responses [17] and enhanced production and activity of immunosuppressive such as IL-10. IL-10 has been shown to be produced by macrophages infected with mycobacteria. IL-10 and TGF-β overlap with each other in many of their biological effects including, inhibition of T cell proliferation and IFN-γ production [21]. Elevated levels of IL-10 in serum during advanced HIV infection may enhance immune suppression, allowing opportunistic infections [21]. In our studies, we observed that NAC treatment decreased the levels of IL-10 favoring immune activation (Fig 5b).

We demonstrate growth inhibition of intracellular H37Rv *in our in vitro *studies using NAC-treated blood cultures from HIV patients. Furthermore, treatment of blood cultures with NAC modulated the production of cytokines in favor of the host. As described in the model (Fig 6a), our results strongly indicate that the immune cell enhancing and antimycobacterial functions of GSH are important for growth control of H37Rv in blood cultures from healthy and HIV-infected subjects (Fig 6a). Additionally, NAC treatment down-regulated the synthesis of IL-10 and pro-inflammatory cytokines in blood cultures from HIV-infected subjects favoring immune activation (Fig 6b). Current interventions to prevent tuberculosis in areas where TB and HIV are endemic, such as sub-Saharan Africa, have serious limitations. ART is limited by its cost and by its requirement for a sophisticated health care delivery system. Isoniazid chemoprophylaxis has limited efficacy in regions of high TB transmission, particularly in highly susceptible individuals with advanced HIV infection. In addition, isoniazid is ineffective against INH-resistant TB strains, which may account for 10–20% of all cases in some areas. NAC is inexpensive and non-toxic (it is considered a food supplement in the US, and is available without prescription in health food stores). The findings from this study may lead to long-term research that will be of potential importance for control of TB worldwide.
